# Health literacy skills in type 2 diabetes mellitus outpatients from an university-affiliated hospital in Rio de Janeiro, Brazil

**DOI:** 10.1186/1758-5996-6-126

**Published:** 2014-11-22

**Authors:** Simone H de Castro, Gilberto N O Brito, Marilia B Gomes

**Affiliations:** Departamento de Medicina Interna, Disciplina de Diabetes, Universidade do Estado do Rio de Janeiro, Rio de Janeiro, RJ Brasil; Departamento de Psiquiatria e Saúde Mental, Instituto de Saúde da Comunidade, Universidade Federal Fluminense, Niterói, RJ Brasil; Departamento de Pesquisa, Instituto Fernandes Figueira, FIOCRUZ, Rio de Janeiro, RJ Brasil; Serviço de Diabetes e Metabologia, Hospital Universitário Pedro Ernesto, Universidade do Estado do Rio de Janeiro, Av 28 setembro 77, 3 andar, 20551-030 Rio de Janeiro, RJ Brasil

**Keywords:** Health literacy, s-TOFHLA, Type 2 diabetes

## Abstract

**Background:**

Type 2 diabetes mellitus is the most common metabolic disorder and has considerable impact on quality of life. Treatment of DM2 is complex and adherence to treatment requires sophisticated cognition which includes literacy skills.

**Methods:**

Health literacy skills of a cross-sectional nonrandom sample of 164 DM2 outpatients at the Diabetes Unit of the Hospital Universitário Pedro Ernesto at the State University of Rio de Janeiro were evaluated by the short version of the Test of Functional Health Literacy in Adults (s-TOFHLA). Procedures available in the SPSS package were used in data analysis.

**Results:**

Fourteen out of 164 patients (8.5%) were completely illiterate and therefore were not further assessed. The remaining 150 patients (75 men and 75 women) were the participants of this study. Data showed that 110 (73.3%) participants had adequate health literacy skills, 17 (11.3%) had marginal skills and 23 (15.3%) had inadequate skills. Moreover, older participants performed worse than younger patients. In addition, Caucasian and multiethnic participants performed better than Afro-Brazilians. Furthermore, participants with higher educational and occupational levels outperformed those with lower levels. However, only age and education, but not ethnic group and occupation, contributed significantly and independently to health literacy.

**Conclusion:**

This study showed that almost a quarter of the participants are illiterate or have inadequate health literacy skills. Therefore, our results indicate the need for the development of health care instructions properly calibrated to the health literacy skills of DM2 patients.

## Background

Type 2 diabetes mellitus (DM2) is the most common metabolic disease with high morbidity and mortality and therefore has important implications for quality of life. Moreover, the incidence and prevalence of DM2 has been steadily increasing and thus this disease has received considerable attention by researchers and health authorities worldwide [1]. In addition, chronic complications of DM2 represent a great burden not only to patients but also to society. The pathophysiology of DM2 complications is certainly complex and derives mostly from chronic hyperglycemia and oxidative stress [2].

The UK Prospective Diabetes Study (1998) showed that adequate management of DM2 significantly reduces the risk of microvascular complications (retinopathy, nephropathy, and possibly neuropathy), the risk of myocardial infarction - fatal and nonfatal - and sudden death by 16% [3]. However, an adequate management of DM2 depends entirely on patients and their families. A major factor in adherence to treatment is the degree of comprehension of disease processes (diabetes knowledge) and the understanding of the treatment protocol itself. Low adherence to treatment seems to adversely affect the clinical outcome and quality of life [3].

Data from the Instituto Brasileiro de Geografia e Estatística (IBGE) indicated that Brazil has 9.07 million illiterates (6.75%) above the age of 17 years [4]. In addition, a survey conducted by the Instituto Paulo Montenegro (IPM) in 2011 showed that 27% of the population between 15 and 64 years of age were functionally illiterate [5]. Therefore, illiteracy and rudimentary literacy are a major public health issue in this country.

DM2 patients with limited literacy skills probably have difficulty to follow prescriptions, instructions for health care and to use educational texts to increase their knowledge of the disease. Such patients have been reported to be more prone to poor glycemic control [6] and the chronic complications of the disease [7, 8]. However, Al Sayah and colleagues in their recent review [9] concluded that the evidence for the relationship between health literacy or numeracy and clinical outcomes is not at all clearcut. Additionally, and importantly, they also reported that health literacy is consistently associated with diabetes knowledge.

Given the importance of literacy skills to patient’s knowledge of diabetes [9] and the lack of information on literacy and numeracy skills of DM2 patients in Brasil, the present study was conducted to assess the frequency of full and functional health illiteracy of DM2 outpatients attended at the Diabetes Unit of the Hospital Universitário Pedro Ernesto (HUPE) at the State University of Rio de Janeiro (UERJ).

## Methods

### Participants

A cross-sectional nonrandom sample of one hundred and sixty four type 2 diabetes mellitus outpatients, 78 men and 86 women, with a mean age of 58.5 yrs (SD = 9.8) and a mean disease duration of 12.9 yrs (SD = 9.5) who came for routine visits to the Diabetes Unit of HUPE were the subjects of this study. Each participant was formally requested to participate and interviewed while waiting for their scheduled routine appointments after his (her) agreement was obtained. Only patients with age ranging from 18 to 80 yrs were included in the study. Patients with hypoglycemia (capillary glycemia <70 mg/dL) at interview and those with limited visual acuity as determined by the inability to read 36-size letters were excluded from the study. The selection of female and male participants was balanced. Ethnicity was assigned solely on the basis of visual inspection of the patient into Caucasian (N = 43, 26.2%), Afro-Brazilian (N = 57, 34.8%) and multiethnic (N = 64, 39.0%) [10]. Fourteen (8.5%) patients were illiterate, 74 (45.1%) had barely completed elementary school, 61 (37.2%) were at the secondary level of education and 15 (9.1%) had been to college. Occupational levels were as described by Hollingshead and Redlich [11]. Briefly, occupational levels 1 and 2 (N = 4, 2.4%) include university-educated executives, business managers in large concerns, and major and lesser professionals; occupational levels 3 (N = 29, 17.7%) and 4 (N = 32, 19.5%) comprise administrative personnel, owners of small independent businesses and minor professionals; and occupational levels 5, 6 and 7 (N = 99, 60.4%) include skilled, semiskilled and unskilled laborers with lower educational levels.

### Instrument

An adaptation of the short version of the Test of Functional Health Literacy in Adults (s-TOFHLA) [12] for the Brazilian population [13] was used to assess the ability of patients to comprehend health care instructions (literacy) and to use quantitative information (numeracy). There was no time limit for completing the test because the objective of the study was to evaluate literacy/numeracy skills without time constraints. The health literacy of the participants was classified as inadequate, marginal or adequate following the scheme reported by Baker and colleagues [12].

The study was conducted in accordance with the Declaration of Helsinki (and subsequent revision) and approved by the Ethics in Research Committee of the HUPE at UERJ.

### Consent

Written informed consent was obtained from the patient for the publication of this report.

### Statistics

Statistical procedures available in the SPSS package (version 20.0, Chicago, Illinois) were used in data analysis.

## Results

After the exclusion of 14 illiterate patients, the analysis of the data for the remaining 150 participants demonstrated that there were no significant differences between men and women in age and s-TOFHLA scores. Therefore, their data were collapsed for additional statistical analyses.

We found that 110 participants (73.3%) had an adequate level of health literacy according to their s-TOFHLA scores, 17 (11.3%) had marginal skills and 23 (15.3%) had inadequate skills. The number of illiterate patients plus the number of participants with inadequate literacy reached 37 or 22.6% of the sample.

Table 1 indicates the proportion of participants in each category of health literacy across age, ethnicity, education and occupation. The proportion of participants with inadequate literacy increased dramatically with age. Furthermore, almost a quarter of Afro-Brazilians had inadequate health literacy skills, but none of the participants with at least a college education was classified as inadequate or marginal literates. Moreover, the frequency of adequate literacy increased the higher the occupational level of the patient.

**Table 1:** **s-TOFHLA results according to age, ethnicity, education and occupation**

	N	Health literacy (N, %)		
		Inadequate	Marginal	Adequate
Age (years)				
≤ 45	21	0 (0)	2 (9.5)	19 (90.5)
46 – 60	62	8 (12.9)	6 (9.7)	48 (77.4)
≥ 61	67	15 (22.4)	9 (13.4)	43 (64.2)
Ethnicity				
Caucasian	42	3 (7.1)	2 (4.8)	37 (88.1)
Afro-Brazilian	55	13 (23.6)	7 (12.7)	35 (63.7)
Multi	53	7 (13.2)	8 (15.1)	38 (71,7)
Education				
Less than 10 yrs	74	21 (28.4)	12 (16.2)	41 (55.4)
10 to 12 yrs	61	2 (3.3)	5 (8.2)	54 (88.5)
College or higher	15	0 (0)	0 (0)	15 (100)
Occupation				
1 and 2	4	0 (0)	0 (0)	4 (100)
3	27	2 (7.4)	1 (3.7)	24 (88.9)
4	31	3 (9.7)	2 (6.5)	26 (83.8)
5 and 6	22	2 (9.1)	5 (22.7)	15 (68.2)
7	66	16 (24.2)	9 (13.7)	41 (62.1)

Table 2 shows median TOFHLA scores across age, ethnic, educational and occupational level groups. Kruskal-Wallis tests revealed significant differences for age such that older participants performed worse than younger participants. In addition, the data demonstrated that Caucasian and multiethnic participants performed better than Afro-Brazilians. Moreover, the higher the educational and occupational levels of the participant the better his (or her) performance on the s-TOFHLA. Further analysis of the data revealed that age, education and occupation were related with both literacy and numeracy, and ethnicity was associated only with literacy skills.

**Table 2:** **Median (range) literacy, numeracy and total s-TOFHLA scores across age, ethnicity, education and occupation**

	Literacy	Numeracy	Total s-OFHLA	N
Age (yrs)				
≤ 45	66 (34 – 72)	21 (14 – 28)	91 (55 – 100)	21
46 – 60	64 (22 – 72)	21 (0 – 28)	85.5 (29 – 100)	62
≥ 61	56 (0 – 72)	21 (7 – 28)	78 (9 – 100)	67
p	0.01	0.05	0.003	
Ethnicity				
Caucasian	68 (22 – 72)	21 (7 – 28)	86 (29 – 100)	42
Afro-Brazilian	56 (0 – 72)	21 (0 – 28)	76 (9 – 100)	55
Multi	64 (20 – 72)	21 (7 – 28)	85 (27 – 100)	53
p	0.004	0.155	0.012	
Education				
Less than 10 yrs	51 (0 – 72)	21 (0 – 28)	70 (9 – 100)	74
10 to 12 yrs	66 (22 – 72)	21 (7 – 28)	87 (29 – 100)	61
College or higher	72 (62 – 72)	28 (7 – 28)	96 (77 – 100)	15
P	< 0.0001	< 0.0001	< 0.0001	
Occupation				
1 and 2	70 (64 – 72)	28 (28 – 28)	98 (92 – 100)	4
3	68 (22 – 72)	21 (7 – 28)	89 (29 – 100)	27
4	68 (30 – 72)	14 (0 – 28)	84 (32 – 100)	31
5 and 6	57 (22 – 72)	21 (7 – 28)	78 (29 – 100)	22
7	53 (0 – 72)	21 (7 – 28)	75 (9 – 100)	66
P	< 0.0001	0.02	< 0.0001	

p levels refer to Kruskal-Wallis test statistics.

A multiple regression performed on the data showed that age, ethnic group, education and occupation significantly predicted s-TOFHLA scores (F_4,145_ = 13.6, p < 0.0001, R^2^ = .273). However, only age and education added significantly to the prediction (p < .032 and p < 0.0001, respectively).

## Discussion

The present study showed that a substantial proportion of the participants were complete illiterates or had inadequate or marginal health literacy skills. Additionally, this report demonstrated significant age effects on health literacy skills such that older individuals performed the s-TOFHLA more poorly than younger people. Moreover, individuals with higher educational and occupational levels outperformed those with more limited education and lower occupational levels and Caucasian and multiethnic participants performed better than Afro-Brazilians. However, only age and education, but not ethnicity and occupation, contributed significantly to s-TOFHLA performance.

It is of interest to note that our data on illiteracy and inadequate health literacy are not far from data obtained in Brazilian national literacy surveys conducted with other assessment instruments. For instance, the comparison of the data herein presented with those derived from the National Demographic Survey conducted in 2010 [4] for individuals with age equal or above 18 yrs showed that the participants of our study had a slightly higher level of complete illiteracy (8.5% vs 6.8%). Also, the percentage of complete illiterates plus inadequate literates in our study (22.6%) was slightly lower than the corresponding figure found by the Indice de Analfabetismo Funcional (INAF) in Brazilians with ages ranging from 15 to 64 yrs (27%) [5]. However, if marginal literates are included, the proportion of illiterates, inadequate and marginal literates in our study would reach 32.9%, i.e., slightly higher than that found by the INAF report [5].

Health literacy studies conducted with the s-TOFHLA have provided discrepant rates of adequate literacy. Schillinger and colleagues [8] found that only 49% of the patients in their study had adequate literacy whereas Kim and collaborators [14] showed that 77% of the participants of their study had adequate literacy. In Brazil, it has been reported that 67.6% of a large sample of healthy participants had adequate health literacy [13]. Therefore, the results from our study are similar to the data presented by the latter two research groups. However, it is important to note that our study was conducted with no time constraints on performance of the s-TOFHLA and in this limited sense provided a more accurate estimate of literacy skills.

Our results that age and education had a significant association with health literacy skills are consistent with data presented elsewhere [15] in the sense that older participants and individuals with lower educational levels had higher levels of inadequate health literacy. Additionally, our results that age, education and occupation were related with both literacy and numeracy add to the evidence on health literacy skills, demography and socioeconomic status. Although our study also showed that ethnicity and occupation had a significant association with literacy skills as determined by nonparametric analysis of variance, a multiple regression analysis indicated that these variables did not contribute independently to s-TOFHLA scores.

Health literacy (literacy and numeracy) denotes a complex set of cognitive and social skills that allow the patients to successfully manage their own health care and include the planning, development and execution of strategies best suited to follow the ever more complicated treatment protocols used in DM2. In addition, adequate health literacy is necessary for the understanding of the information provided by the health care system to increase diabetes knowledge and adherence to treatment. It seems clear that the cognitive demands placed on DM2 patients for an adequate disease management are by no means simple and therefore it should not be surprising that adherence to treatment is so limited [16].

There are limitations in our study. Firstly, we did not impose time constraints on the performance of the s-TOFHLA and therefore our patients did not have the added pressure of time to complete the tasks. Secondly, this instrument evaluates only health literacy based on literacy (reading) and numeracy (quantitative information) skills.

A model to determine the path from health literacy to clinical outcomes which includes abilities like knowledge, self-monitoring, attitudes, initiation and adherence to health behavior among others has been proposed [17]. It is interesting to note that these abilities probably represent fundamental neurobehavioral dimensions as, for example, affective, memory, language processes and especially cognitive flexibility and other executive functions. It is therefore important to investigate the association of neurobehavioral dimensions with clinical outcomes in DM2 patients. Research along these lines is currently underway in the Neurocognitive Diabetology Group of our unit.

## Conclusions

We have shown that a substantial proportion of patients seen in our Diabetes Unit are illiterate or have inadequate health literacy skills. Since poorer performance on the s-TOFHLA is mostly seen in older, less educated patients with lower occupational levels, and considering that a major part of our patients have such features, it is important that treatment and information protocols be carefully calibrated to reach the goal of providing adequate health care to our patient population.
